# Change in Anemia by Carnitine Supplementation in Patients Undergoing Peritoneal Dialysis: A Retrospective Observational Study

**DOI:** 10.3389/fmed.2021.767945

**Published:** 2021-11-05

**Authors:** Shohei Kaneko, Katsunori Yanai, Taisuke Kitano, Haruhisa Miyazawa, Keiji Hirai, Susumu Ookawara, Yoshiyuki Morishita

**Affiliations:** Division of Nephrology, First Department of Integrated Medicine, Saitama Medical Center, Jichi Medical University, Saitama, Japan

**Keywords:** carnitine deficiency, carnitine supplementation, peritoneal dialysis, erythropoietin resistance index, erythropoietin-resistant anemia

## Abstract

**Background:** Carnitine supplementation improves various dialysis-related symptoms including erythropoietin-resistant anemia in patients who are undergoing hemodialysis. However, the utility of carnitine supplementation in patients who are undergoing peritoneal dialysis (PD) is not fully understood.

**Methods:** Thirteen patients undergoing PD [mean age: 54.2 ± 14.8 years, males: 9/13 (69%)] administered oral carnitine supplementation (mean dose: 9.1 ± 3.3 mg/kg/day) for 4–6 months were retrospectively investigated. Changes in serum carnitine levels and other clinical variables including the erythropoietin resistance index (ERI) were analyzed after carnitine supplementation.

**Results:** Carnitine supplementation increased serum total carnitine (48.5 ± 10.2 vs. 130.1 ± 37.2 μmol/L, *P* < 0.01), free carnitine (31.1 ± 8.3 vs. 83.1 ± 24.6 μmol/L, *P* < 0.01), and acyl carnitine (17.4 ± 2.8 vs. 46.9 ± 13.8, *P* < 0.01) levels. The acyl carnitine/free carnitine ratio was not affected (0.6 ± 0.1 vs. 0.6 ± 0.1, *P* = 0.75). Although the mean ERI was not affected by carnitine supplementation [13.7 ± 4.7 vs. 11.6 ± 3.4 IU/kg/(g/dL)/week, *P* = 0.28], the ERI change rate was significantly decreased (1.00 ± 0.00 vs. 0.87 ± 0.11, *P* < 0.01).

**Conclusion:** Carnitine supplementation may improve erythropoietin resistance in patients who are undergoing PD.

## Introduction

Carnitine is a water-soluble amino acid derivative with a molecular weight of 161 Da, and it has an important role in fatty acid metabolism in skeletal muscle (1–3). Seventy-five percent of the human body requirement for carnitine is obtained from the diet such as red meat, while the remaining 25% is biosynthesized in the kidneys and liver (4, 5). Carnitine exists in different forms in serum including free carnitine and acyl carnitine (6, 7). Total carnitine is the sum of free carnitine and acyl carnitine (6, 7). Free carnitine is converted to acyl carnitine by binding acyl residues, and it can transfer acyl residues into mitochondria for use as an energy source (1, 2). However, excess and harmful acyl residues are also discharged as acyl carnitines (1, 2). Therefore, to maintain homeostasis of energy metabolism, the different forms of carnitine must be properly balanced.

Low serum free carnitine and/or high acyl carnitine/free carnitine ratio generally result in diagnosis of carnitine deficiency (8). In the healthy population, the frequency of carnitine deficiency is very low because carnitine is supplied satisfactorily through diet and renal and hepatic biosynthesis (4, 5). However, carnitine deficiency is frequently observed in patients undergoing hemodialysis (4, 9, 10). Possible mechanisms for the deficiency include dietary restrictions attributed to renal failure, decreased biosynthesis, and exposure to dialysate (11, 12). Previous studies have reported a loss of more than 60% of free carnitine in one hemodialysis session (13). Additionally, a Japanese research group recently reported that hemodiafiltration contributed more to carnitine loss than hemodialysis (hemodialysis 64% ± 4% vs. hemodiafiltration 75% ± 7%) (10). Typically, carnitine deficiency is associated with various symptoms such as erythropoietin-resistant anemia, muscle weakness, cardiac dysfunction, and intradialytic hypotension (14). Additionally, recent studies have suggested an association between carnitine deficiency and conditions such as glucose metabolism disorders and liver dysfunction (15–18). National Kidney Foundation guidelines recommend carnitine supplementation for patients with carnitine deficiency undergoing hemodialysis (14).

However, at this time, only a few reports have investigated carnitine deficiency in patients undergoing peritoneal dialysis (PD) (19–21). Clinicians should note that the National Kidney Foundation guidelines are not sufficiently supported by reports of carnitine deficiency in patients undergoing PD (14). Under these circumstances, we previously reported a high prevalence of carnitine deficiency in patients undergoing PD in a cross-sectional study (21). Additionally, we found a significant association between the acyl carnitine/free carnitine ratio and the erythropoietin resistance index (ERI) in this population (21). The ERI is an indicator for the severity of erythropoietin-resistant anemia (22, 23), and it is associated with the mortality rate in patients undergoing dialysis (23). Therefore, carnitine deficiency is considered to be an important pathological condition in patients undergoing hemodialysis and in patients undergoing PD. The utility of carnitine supplementation in the patients undergoing PD remains unclear because, to date, it has not been fully investigated. Therefore, in the present study, we retrospectively investigated the effects of carnitine supplementation in patients with carnitine deficiency who were undergoing PD.

## Materials and Methods

### Study Design

This was a single-center, retrospective, and observational study. We compared serum carnitine concentrations (free carnitine, acyl carnitine, and total carnitine) and other clinical variables before and after 4–6 months of oral carnitine supplementation.

### Study Population

This study was conducted at Saitama Medical Center, Jichi Medical University between September 2016 and August 2021. We included patients undergoing PD who were administered carnitine for 4–6 months to treat carnitine deficiency. Inclusion criteria were as follows: (i) patients undergoing PD diagnosed with carnitine deficiency (based on criteria discussed below); (ii) patients supplemented with carnitine for 4–6 months; (iii) patients whose carnitine concentrations were measured before and after carnitine supplementation; and (iv) age > 18 years. Exclusion criteria were as follows: (i) patients with acute kidney injury; (ii) patients who underwent renal transplantation; (iii) patients undergoing additional hemodialysis; and (iv) patients who refused to participate in the study. Patients who were diagnosed with carnitine deficiency and did not receive carnitine supplementation were designated as the control group. The control group was followed-up for 6 months, which included evaluation of clinical variables. All patients undergoing PD were treated according to the 2009 Japanese Society for Dialysis Therapy Guideline for PD and 2015 Japanese Society for Dialysis Therapy Guideline for Renal Anemia in Chronic Kidney Disease (24, 25). This study was approved by the Saitama Medical Center, Jichi Medical University Ethics Committee (DAI-RIN 15-34) and was conducted in accordance with the Declaration of Helsinki. Patients were notified of their option to opt out of the study.

### Data Collection

We obtained patient data from medical records. These included age, sex, body mass index, systolic blood pressure, diastolic blood pressure, comorbidities (diabetes mellitus, hepatic diseases, cardiac diseases, malignant tumor and bone marrow disease), Administration renin-angiotensin inhibitor, Amount of PD fluid, Amount of water removal, Amount of urine, Energy intake, Protein intake, PD period, PD modalities, dialysis efficiency (weekly Kt/V urea, peritoneal Kt/V urea, and renal Kt/V urea), 4-h dialysate/plasma creatinine, ejection fraction, cardio-thoracic ratio, blood tests, and dialysate tests. PD modalities were categorized as either continuous ambulatory peritoneal dialysis (CAPD) or automated peritoneal dialysis (APD). CAPD is a method whereby patients manually change the dialysate, whereas in APD, the dialysate is changed automatically by an automated cycler (26–29). Peritoneal weekly Kt/V urea was used as indicator of efficacy of PD, and renal weekly Kt/V was used as indicator of residual renal function (30). Their summation was defined as total weekly Kt/V urea (30). 4-h dialysate/plasma creatinine is an indicator of permeability of peritoneal membranes (31). Ejection fraction was measured by echocardiography. The Cardio-thoracic ratio was measured by chest X-ray. Serum total carnitine and free carnitine concentrations were determined at a clinical chemistry laboratory (SRL, Tokyo, Japan). They were measured using the enzyme cycling method (6). Acyl carnitine concentrations were calculated as total carnitine minus free carnitine (7). Blood tests (except for carnitine and dialysate tests) were performed by the Department of Clinical Laboratory, Saitama Medical Center, Jichi Medical University.

### Definition of Carnitine Deficiency

Diagnosis of carnitine deficiency was based on Japanese guidelines (low free carnitine (<36 μmol/L) and/or high acyl carnitine/free carnitine ratio (>0.4)) (8). This guideline was published by the Japan Pediatric Society in 2018 (8).

### Carnitine Supplementation

Japanese guidelines recommend carnitine supplementation at doses of 10–20 mg/body weight (kg) in patients undergoing PD diagnosed with carnitine deficiency (8). However, this dose was not supported by previous reports. Dosage was determined based on these guidelines and the tolerability of patients. After 4–6 months, serum carnitine concentrations (free carnitine, acyl carnitine, and total carnitine) were remeasured.

### Erythropoietin Resistance Index

ERI was used as indicator of resistance of renal anemia to erythropoietin-stimulating agents (22, 23). ERI was defined as average weekly dose of recombinant human erythropoietin (IU)/body weight (kg)/hemoglobin (g/dL) (22, 23). For dose conversion of recombinant human erythropoietin, the ratio of recombinant human erythropoietin: darbepoetin alfa or epoetin beta pegol was converted to 200:1 (32). Changes in ERI in individual patients were analyzed as the ERI change rate. The ERI change rate was calculated as follows: ERI (after carnitine supplementation) / ERI (baseline).

### Statistical Analysis

Quantitative variables are presented as mean ± standard deviation. Categorical variables are presented as frequency and percentage. Continuous variables were compared using the paired *t*-test. Frequency was compared using Fisher's exact test. Statistical analyses were performed using JMP (SAS Institute Inc., Cary, NC, USA). *P* < 0.05 was considered statistically significant.

## Results

### Clinical Features of the Study Population

There were 13 patients in the treatment group and 13 patients in the control group who met the study entry criteria. Features of the study population are shown in Table 1. In the treatment group, the mean age was 54.2 ± 14.8 years, and nine of 13 patients were men (69%). The mean body mass index was 22.2 ± 3.0 kg/m^2^. The mean PD period was 28.9 ± 19.8 months. Five (38%) patients underwent CAPD, while eight (62%) patients underwent APD. The mean total Kt/V urea, peritoneal Kt/V urea, and renal Kt/V urea were 1.6 ± 0.3, 1.3 ± 0.2, and 0.3 ± 0.3, respectively. The mean carnitine supplementation dose was 9.1 ± 3.3 mg/kg/day. In the control group, the mean age was 60.3 ± 11.5 years, and nine of 13 patients were men (69%). The mean body mass index was 23.1 ± 2.0 kg/m^2^. The mean PD period was 48.0 ± 36.6 months. A significant difference in the mean ERI between the two groups was observed (13.7 ± 4.7 vs. 6.9 ± 2.6 [IU/kg/(g/dL)/week], *P* < 0.01).

**Table 1:** Features of the study population.

	**Treatment group (*n* = 13)**	**Control group (*n* = 13)**	
**Variable**	**Mean ± SD or number (%)**	**Mean ± SD or number (%)**	***P*-Value**
Age (years)	54.2 ± 14.8	60.3 ± 11.5	0.27
Male sex	9 (69)	9 (69)	1.00
Body mass index (kg/m^2^)	22.2 ± 3.0	23.1 ± 2.0	0.42
Systolic blood pressure (mmHg)	148.4 ± 9.5	154.2 ± 22.6	0.42
Diastolic blood pressure (mmHg)	84.6 ± 10.6	87.7 ± 10.6	0.48
Diabetes mellitus	2 (15)	5 (38)	0.20
Hepatic disease	2 (15)	1 (8)	0.56
Cardiac disease	3 (23)	6 (46)	0.23
Malignant tumor	1 (8)	0 (0)	1.00
Bone marrow disease	1 (8)	0 (0)	1.00
Administration renin-angiotensin inhibitor	9 (69)	10 (77)	1.00
Amount of PD fluid (mL/day)	6861.5 ± 2099.6	5955.4 ± 1786.5	0.27
Amount of water removal (mL/day)	853.4 ± 640.3	703.8 ± 509.3	0.53
Amount of urine (mL/day)	634.6 ± 503.3	669.2 ± 582.2	0.88
Energy intake (kcal/kg/day)	26.1 ± 7.1	26.0 ± 4.2	0.96
Protein intake (g/kg/day)	0.8 ± 0.3	0.9 ± 0.2	0.29
PD period (months)	28.9 ± 19.8	48.0 ± 36.6	0.13
Hemodialysis combination	0 (0)	0 (0)	1.00
PD modality: CAPD	5 (38)	7 (54)	0.45
PD modality: APD	8 (62)	6 (46)	0.45
Total Kt/V urea	1.6 ± 0.3	1.5 ± 0.5	0.49
Peritoneal Kt/V urea	1.3 ± 0.2	1.1 ± 0.4	0.22
Renal Kt/V urea	0.3 ± 0.3	0.5 ± 0.3	0.11
D/P Creatinine	0.7 ± 0.1	0.7 ± 0.1	0.49
Dose of carnitine supplementation (mg/kg/day)	9.1 ± 3.3	(Un-administered)	-
White blood cells (10^3^/μL)	5.2 ± 1.7	6.1 ± 1.0	0.15
Red blood cells (10^4^/μL)	329.4 ± 47.9	347.8 ± 43.9	0.33
Hemoglobin (g/dL)	9.9 ± 1.2	10.7 ± 1.4	0.15
Platelets (× 10^4^/μL)	17.4 ± 5.7	20.3 ± 5.8	0.23
Total protein (g/dL)	6.1 ± 0.4	6.2 ± 0.5	0.66
Albumin (g/dL)	3.3 ± 0.4	3.4 ± 0.5	0.55
Blood urea nitrogen (mg/dL)	58.6 ± 6.8	60.2 ± 17.4	0.78
Creatinine (mg/dL)	13.5 ± 2.7	11.7 ± 1.6	0.05
Aspartate aminotransferase (IU/L)	12.0 ± 7.8	14.4 ± 3.4	0.34
Alanine aminotransferase (IU/L)	13.1 ± 9.1	13.5 ± 5.5	0.88
γ-glutamyl transpeptidase (IU/L)	16.9 ± 8.3	23.0 ± 21.1	0.36
Alkaline phosphatase (IU/L)	330.2 ± 191.6	260.4 ± 118.0	0.29
C-reactive protein (mg/dL)	0.2 ± 0.3	0.2 ± 0.2	0.72
Uric acid (mg/dL)	5.7 ± 1.0	6.6 ± 1.4	0.09
Sodium (mEq/L)	136.0 ± 2.9	138.0 ± 3.4	0.12
Potassium (mEq/L)	4.7 ± 0.5	4.4 ± 0.8	0.27
Chloride (mEq/L)	97.4 ± 4.9	99.3 ± 3.9	0.30
Corrected calcium (mg/dL)	9.4 ± 0.6	9.2 ± 0.8	0.39
Phosphorus (mg/dL)	5.5 ± 1.4	5.7 ± 1.3	0.71
Magnesium (mg/dL)	2.0 ± 0.2	2.2 ± 0.4	0.13
Zinc (μg/dL)	71.3 ± 20.6	59.6 ± 7.94	0.09
Iron (μg/dL)	123.0 ± 40.2	91.8 ± 38.4	0.07
Ferritin (mg/mL)	202.8 ± 113.1	202.1 ± 147.7	0.99
Transferrin saturation (%)	47.1 ± 11.4	35.2 ± 15.4	0.06
Vitamin B12 (pg/mL)	653.0 ± 330.1	513.5 ± 183.5	0.65
Folate (ng/mL)	4.5 ± 1.4	3.2 ± 0.6	0.38
Blood sugar (mg/dL)	111.0 ± 18.4	114.6 ± 22.1	0.67
HbA1c (%)	5.2 ± 0.3	5.3 ± 0.5	0.45
Total carnitine (μmol/L)	48.5 ± 10.2	45.7 ± 14.5	0.60
Acyl carnitine (μmol/L)	17.4 ± 2.8	18.5 ± 6.8	0.60
Free carnitine (μmol/L)	31.1 ± 8.3	27.2 ± 8.7	0.28
Acyl carnitine/free carnitine ratio	0.6 ± 0.2	0.7 ± 0.2	0.13
Total cholesterol (mg/dL)	180.8 ± 30.8	177.8 ± 46.6	0.85
HDL cholesterol (mg/dL)	52.8 ± 13.5	49.3 ± 9.7	0.48
LDL cholesterol (mg/dL)	103.3 ± 23.7	100.4 ± 42.3	0.84
Triglycerides (mg/dL)	113.2 ± 39.2	114.2 ± 42.9	0.95
Intact PTH (pg/dL)	226.1 ± 135.8	229.2 ± 120.8	0.95
ERI [IU/Kg/(g/dL)/week]	13.7 ± 4.7	6.9 ± 2.6	<0.01
Ejection fraction (%)	60.1 ± 8.4	60.2 ± 13.2	0.98
Cardio-thoracic ratio (%)	48.3 ± 5.2	50.4 ± 5.5	0.35

*SD, standard deviation; PD, peritoneal dialysis; CAPD, continuous ambulatory peritoneal dialysis; APD, automated peritoneal dialysis; D/P Creatinine, 4-h dialysate/plasma creatinine ratio; HbA1c, hemoglobin A1c; HDL, high-density lipoprotein; LDL, low-density lipoprotein; PTH, parathormone; ERI, erythropoietin resistance index*.

### Effects of Carnitine Supplementation on Carnitine Concentrations

Changes in carnitine concentrations in response to carnitine supplementation are shown in Table 2. Carnitine supplementation increased serum total carnitine (48.5 ± 10.2 vs. 130.1 ± 37.2 μmol/L, *P* < 0.01), free carnitine (31.1 ± 8.3 vs. 83.1 ± 24.6 μmol/L, *P* < 0.01), and acyl carnitine (17.4 ± 2.8 vs. 46.9 ± 13.8, *P* < 0.01). The acyl carnitine/free carnitine ratio was not affected by carnitine supplementation (0.6 ± 0.1 vs. 0.6 ± 0.1, *P* = 0.75).

**Table 2:** Effects of carnitine supplementation on clinical variables.

	**Treatment group (*****n*** **=** **13)**			**Control group (*****n*** **=** **13)**		
**Variable**	**Baseline**	**After carnitine supplementation**	***P*-Value**	**Baseline**	**After follow-up**	***P*-Value**
Systolic blood pressure (mmHg)	148.4 ± 9.5	148.7 ± 16.2	0.96	154.2 ± 22.6	153.8 ± 22.9	0.97
Diastolic blood pressure (mmHg)	84.6 ± 10.6	86.7 ± 9.4	0.62	87.7 ± 10.6	80.4 ± 14.6	0.17
White blood cells (10^3^/μL)	5.2 ± 1.7	5.3 ± 1.4	0.89	6.1 ± 1.0	5.7 ± 1.4	0.48
Red blood cells (10^4^/μL)	329.4 ± 47.9	330.6 ± 43.8	0.95	347.8 ± 43.9	350.3 ± 30.9	0.88
Hemoglobin (g/dL)	9.9 ± 1.2	10.1 ± 1.2	0.78	10.7 ± 1.4	10.4 ± 1.0	0.47
Platelets (× 10^4^/μL)	17.4 ± 5.7	18.5 ± 7.8	0.70	20.3 ± 5.8	18.6 ± 5.5	0.45
Total protein (g/dL)	6.1 ± 0.4	6.1 ± 0.4	0.90	6.2 ± 0.5	5.9 ± 0.4	0.10
Albumin (g/dL)	3.3 ± 0.4	3.2 ± 0.3	0.49	3.4 ± 0.5	3.1 ± 0.5	0.10
Blood urea nitrogen (mg/dL)	58.6 ± 6.8	60.3 ± 17.2	0.75	60.2 ± 17.4	62.6 ± 14.1	0.71
Creatinine (mg/dL)	13.5 ± 2.7	13.4 ± 3.7	0.72	11.7 ± 1.6	11.8 ± 1.3	0.85
Aspartate aminotransferase (IU/L)	12.0 ± 7.8	12.5 ± 4.7	0.86	14.4 ± 3.4	15.2 ± 5.4	0.68
Alanine aminotransferase (IU/L)	13.1 ± 9.1	13.3 ± 6.2	0.94	13.5 ± 5.5	17.2 ± 9.5	0.25
γ-glutamyl transpeptidase (IU/L)	16.9 ± 8.3	18.2 ± 8.9	0.73	23.0 ± 21.1	23.2 ± 19	0.98
Alkaline phosphatase (IU/L)	330.2 ± 191.6	336.2 ± 159.0	0.93	260.4 ± 118.0	294.0 ± 97.9	0.45
C-reactive protein (mg/dL)	0.2 ± 0.3	0.3 ± 0.3	0.52	0.2 ± 0.2	0.2 ± 0.3	0.92
Uric acid (mg/dL)	5.7 ± 1.0	5.6 ± 0.8	0.80	6.6 ± 1.4	6.4 ± 1.1	0.75
Sodium (mEq/L)	136.0 ± 2.9	137.0 ± 3.2	0.22	138.0 ± 3.4	137.0 ± 2.7	0.65
Potassium (mEq/L)	4.7 ± 0.5	4.7 ± 0.5	0.81	4.4 ± 0.8	4.5 ± 0.7	0.90
Chloride (mEq/L)	97.4 ± 4.9	98.9 ± 4.2	0.43	99.3 ± 3.9	99.2 ± 3.0	0.91
Corrected calcium (mg/dL)	9.4 ± 0.6	9.0 ± 0.6	0.10	9.2 ± 0.8	9.4 ± 0.5	0.38
Phosphorus (mg/dL)	5.5 ± 1.4	5.8 ± 1.2	0.52	5.7 ± 1.3	5.6 ± 1.2	0.86
Magnesium (mg/dL)	2.0 ± 0.2	2.1 ± 0.3	0.52	2.2 ± 0.4	2.1 ± 0.3	0.30
Zinc (μg/dL)	71.3 ± 20.6	73.0 ± 20.6	0.87	59.6 ± 7.94	61.2 ± 11.8	0.72
Iron (μg/dL)	123.0 ± 40.2	93.2 ± 29.2	0.05	91.8 ± 38.4	86.7 ± 34.8	0.73
Ferritin (mg/mL)	202.8 ± 113.1	142.5 ± 83.8	0.17	202.1 ± 147.7	173.9 ± 100.8	0.59
Transferrin saturation (%)	47.1 ± 11.4	37.5 ± 11.5	0.05	35.2 ± 15.4	34.6 ± 13.5	0.92
Vitamin B12 (pg/mL)	653.0 ± 330.1	810.7 ± 272.5	0.50	513.5 ± 183.5	66.4 ± 133.1	0.35
Folate (ng/mL)	4.5 ± 1.4	3.8 ± 0.9	0.57	3.2 ± 0.6	4.1 ± 1.3	0.46
Blood sugar (mg/dL)	111.0 ± 18.4	113.2 ± 50.8	0.89	114.6 ± 22.1	120.2 ± 28.5	0.60
HbA1c (%)	5.2 ± 0.3	5.3 ± 0.5	0.40	5.3 ± 0.5	5.6 ± 0.6	0.26
Total carnitine (μmol/L)	48.5 ± 10.2	130.1 ± 37.2	<0.01	45.7 ± 14.5	(unmeasured)	-
Acyl carnitine (μmol/L)	17.4 ± 2.8	46.9 ± 13.8	<0.01	18.5 ± 6.8	(unmeasured)	-
Free carnitine (μmol/L)	31.1 ± 8.3	83.1 ± 24.6	<0.01	27.2 ± 8.7	(unmeasured)	-
Acyl carnitine/free carnitine ratio	0.6 ± 0.1	0.6 ± 0.1	0.75	0.7 ± 0.2	(unmeasured)	-
Total cholesterol (mg/dL)	180.8 ± 30.8	188.5 ± 23.9	0.50	177.8 ± 46.6	152.5 ± 38.0	0.17
HDL cholesterol (mg/dL)	52.8 ± 13.5	53.5 ± 11.4	0.88	49.3 ± 9.7	45.0 ± 10.1	0.31
LDL cholesterol (mg/dL)	103.3 ± 23.7	111.8 ± 18.4	0.34	100.4 ± 42.3	80.9 ± 34.7	0.24
Triglycerides (mg/dL)	113.2 ± 39.2	109.9 ± 46.8	0.85	114.2 ± 42.9	114.5 ± 49.5	0.99
Intact PTH (pg/dL)	226.1 ± 135.8	288.2 ± 175.3	0.34	229.2 ± 120.8	194.4 ± 90.4	0.44
ERI [IU/Kg/(g/dL)/week]	13.7 ± 4.7	11.6 ± 3.4	0.28	6.9 ± 2.6	8.1 ± 3.0	0.28
Change rate of ERI	1.00 ± 0.00	0.87 ± 0.11	<0.01	1.00 ± 0.00	1.19 ± 3.0	<0.01
Ejection fraction (%)	60.1 ± 8.4	60.2 ± 13.2	0.98	60.2 ± 13.2	67.0 ± 7.0	0.69
Cardio-thoracic ratio (%)	48.3 ± 5.2	48.7 ± 5.9	0.86	50.4 ± 5.5	51.7 ± 4.1	0.51

*HbA1c, hemoglobin A1c; HDL, high-density lipoprotein; LDL, low-density lipoprotein; PTH, parathormone; ERI, erythropoietin resistance index*.

### Effects of Carnitine Supplementation on ERI

**Figure 1:**
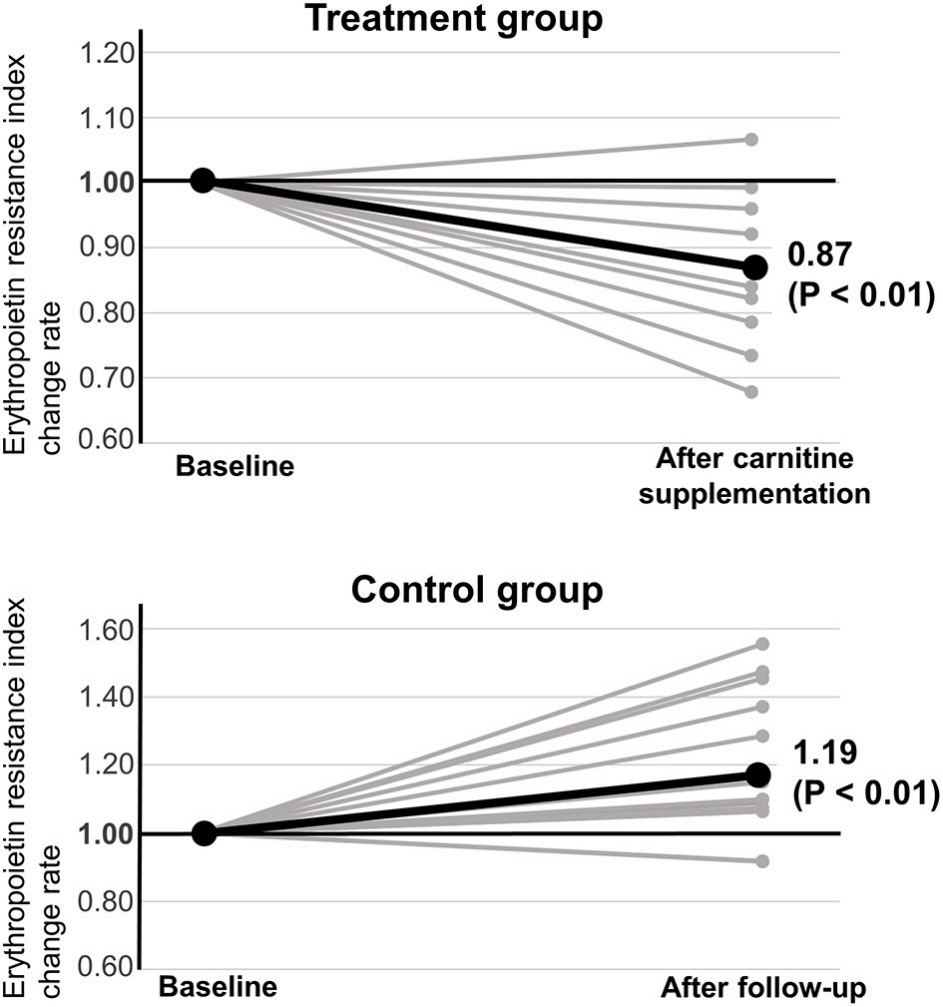
Effect of carnitine supplementation on erythropoietin resistance index.

The effects of carnitine supplementation on clinical variables, including ERI, are shown in Table 2 and Figure 1. In the treatment group, two patients who developed severe infections and one who developed severe anemia of unknown cause were excluded from the ERI analysis. Although oral carnitine supplementation had a tendency to decrease the mean ERI value, there was no significant difference between the two groups [13.7 ± 4.7 vs. 11.6 ± 3.4 IU/kg/(g/dL)/week, *P* = 0.28]. However, the change rate of ERI was significantly decreased by carnitine supplementation (1.00 ± 0.00 vs. 0.87 ± 0.11, *P* < 0.01). In the control group, the mean ERI value did not change (6.9 ± 2.6 vs. 8.1 ± 3.0 IU/kg/(g/dL)/week, *P* = 0.28) and the ERI change rate was significantly increased (1.00 ± 0.00 vs. 1.19 ± 3.0, *P* < 0.01) after 6 months of follow-up. No other clinical parameters were affected by carnitine supplementation in this study.

## Discussion

In this study, 4–6 months of oral carnitine supplementation (mean dose: 9.1 ± 3.3 mg/kg/day) in patients undergoing PD increased total carnitine, free carnitine, and acylcarnitine levels, and did not affect the acyl carnitine/free carnitine ratio. Although carnitine supplementation did not decrease the mean ERI value, the ERI change rate was significantly decreased. In the control group, the mean ERI value was not changed after 6 months of follow-up and the ERI change rate was significantly increased. These results suggest that erythropoietin resistance in patients who are undergoing PD may be improved by oral carnitine supplementation. The difference in the baseline ERI between the two groups (13.7 ± 4.7 vs. 6.9 ± 2.6 [IU/kg/(g/dL)/week], *P* < 0.01) may be because we preferentially administered carnitine to patients with severe erythropoietin resistance in accordance with the guidelines (14). Although this short-term observational study had a small sample size, the results are important because few similar studies have been conducted (19, 20). In Japan, carnitine measurement was covered by public medical insurance starting in February 2018, and we expect that the details on carnitine deficiency in patients who are undergoing PD will be gradually clarified in the future.

In patients undergoing PD, carnitine deficiency can be explained by several factors. First, patients with kidney disease, including those undergoing PD, must follow dietary restrictions. Therefore, oral intake of carnitine is reduced (11, 12). Second, because carnitine is biosynthesized in the kidney, it tends to be deficient in patients with impaired renal function (11, 12). Third, loss of carnitine in PD fluid is also possible (33). Although a previous study showed that APD contributed more to free carnitine loss than CAPD (34), our previous studies did not show the same results (21). Additionally, the hemodialysis combination (daily PD + hemodialysis once a week) did not affect the carnitine level in our previous study (21).

At present, there are few reports on carnitine supplementation in patients undergoing PD (19, 20). The National Kidney Foundation recommends 9–12 months of carnitine supplementation for dialysis patients. However, this is intended for patients undergoing hemodialysis, not those undergoing PD (14). Japanese guidelines propose oral carnitine supplementation at 10–20 mg/kg in patients undergoing PD, although there are no previous reports that support these guidelines. Therefore, the optimal treatment period, supplementation dose, and monitoring frequency for carnitine deficiency in patients who are undergoing PD have not been established. To avoid unexpected adverse effects, we performed a relatively short-term study (4–6 months) using a relatively small dose (9.1 ± 3.3 mg/kg/day) of carnitine.

We previously reported the importance of the acyl carnitine/free carnitine ratio for diagnosing carnitine deficiency in patients undergoing PD (21). In the current study, ERI decreased following carnitine supplementation with increasing levels of both free carnitine (from 17.4 ± 3.0 to 46.9 ± 14.5 μmol/L) and acyl carnitine (from 31.1 ± 8.6 to 83.1 ± 25.8 μmol/L). The acyl carnitine/free carnitine ratio was not changed by carnitine supplementation. According to these results, increased free carnitine level may be more effectives than corrected balance of acyl carnitine/free carnitine ratio for improving ERI in patients undergoing PD. Future studies with larger sample sizes and longer observational periods are required to further clarify the effects and proper monitoring of carnitine supplementation in patients undergoing PD with carnitine deficiency.

Carnitine is considered to be associated with red blood cell deformability (22, 35, 36). Therefore, carnitine deficiency is considered to be one of the factors that caused the increased ERI (21). To our knowledge, there are only two reports on the effects of carnitine supplementation on erythropoietin-resistant anemia in patients undergoing PD (19, 20). Sotirakopoulos et al. reported that anemia improved in 12 patients undergoing PD who received carnitine supplementation (19). In contrast, Lilien et al. reported that carnitine supplementation did not improve anemia in four pediatric patients undergoing PD (20). The differences between these reports and our report may be explained by oral medication adherence. Poor oral medication adherence is often a serious problem in clinical settings. At our institution, we thoroughly checked the oral medication adherence and confirmed that the patients did not miss a dose. Additionally, the effects of carnitine supplementation on erythropoietin-resistant anemia may be associated with differences in the carnitine supplementation dose, supplementation period, and patient background.

This study had important limitations. First, this was an observational study with a small sample size, and additional large-scale studies are needed. Second, it was conducted at a single facility. Third, carnitine supplementation was only administered orally, and carnitine-containing PD fluid was not considered. Fourth, the results may have been affected by differences in carnitine supplementation dose and period. Fifth, this finding cannot be applied to patients without carnitine deficiency. Large-scale interventional studies are required to clarify the effectiveness of carnitine supplementation in patients who are undergoing PD with carnitine deficiency.

## Conclusions

Carnitine supplementation may improve erythropoietin resistance in patients undergoing PD.

## Data Availability Statement

The raw data supporting the conclusions of this article will be made available by the authors, without undue reservation.

## Ethics Statement

The studies involving human participants were reviewed and approved by Saitama Medical Center, Jichi Medical University Ethics Committee (DAI-RIN 15-34). Written informed consent for participation was not required for this study in accordance with the National Legislation and the Institutional requirements.

## Author Contributions

SK: conceptualization, methodology, software, validation, formal analysis, investigation, resources, visualization, and writing—original draft preparation. SK, KY, TK, HM, KH, SO, and YM: data curation. SK and YM: writing—review and editing. YM: supervision and project administration. All authors have read and agreed to the published version of the manuscript.

## Conflict of Interest

The authors declare that the research was conducted in the absence of any commercial or financial relationships that could be construed as a potential conflict of interest.

## Publisher's Note

All claims expressed in this article are solely those of the authors and do not necessarily represent those of their affiliated organizations, or those of the publisher, the editors and the reviewers. Any product that may be evaluated in this article, or claim that may be made by its manufacturer, is not guaranteed or endorsed by the publisher.
